# Bioinspired Functional Design for Wearable Environmental Sensors

**DOI:** 10.3390/biomimetics10100698

**Published:** 2025-10-15

**Authors:** Haejin Bae

**Affiliations:** Ecological Technology Research Team, Division of Restoration Ecology Research, National Institute of Ecology, Seocheon-gun 33657, Chungcheongnam-do, Republic of Korea; hjbae@nie.re.kr

**Keywords:** biomimetics, wearable sensors, adhesion, color change, antifouling, structural color

## Abstract

Biological mechanisms observed across diverse species—such as adhesion, color change, antifouling, and flexible protection—are functionally classified to inform a principle-based conceptual framework for the design of wearable environmental sensors. Existing wearable sensors are constrained by poor skin conformity, reliance on chemical adhesives, performance degradation in wet environments, dependency on external power, and low durability. In response, biological case studies are systematically organized into four functional categories—reversible and wet adhesion, power-free coloration, antifouling and antibacterial surface mechanisms, and compliant protective architectures—and hierarchically mapped to corresponding engineering layers. Rather than reporting experimental results, this framework outlines how biological mechanisms was translated into structured design principles that collectively address the core requirements of wearable sensors: skin compatibility, energy efficiency, fouling resistance, and durability under mechanical deformation. Unlike previous biomimetic surveys that primarily catalog natural phenomena, this work establishes a structured, function-oriented framework that explicitly connects biological strategies to multilayer sensor architectures aligned with Nature-based Solutions and the Global Biodiversity Framework. Ultimately, it clarifies a unique conceptual pathway for sustainable, biodiversity-informed engineering design.

## 1. Introduction

Over the past few decades, accelerated industrialization and urbanization have delivered rapid technological and economic advances while simultaneously precipitating intertwined crises including climate change, environmental pollution, biodiversity loss, and resource depletion [1,2]. These challenges now threaten societal sustainability well beyond the environmental sector. In response, the international community has adopted the Global Biodiversity Framework (GBF) to advance strategies that jointly realize biodiversity conservation and climate action [3]. To translate such strategies into practice, new scientific evidence and innovative approaches are required.

Environmental sensing technologies can play a pivotal role in this context. Sensors that precisely monitor temperature, humidity, air quality, and water quality enable early detection of ecosystem disturbances, inform habitat management and climate impact analyses, and support public-health applications [4,5,6,7]. Following the COVID-19 pandemic, demand has surged for technologies capable of simultaneously monitoring environmental and health-related signals, particularly for skin-mounted wearable sensors that provide real-time information from close proximity to the user [8,9,10]. Such devices are increasingly expected to operate continuously in outdoor environments, to communicate data without bulky peripherals, and to maintain biocompatibility over weeks of use—performance criteria that conventional electronic platforms rarely achieve without trade-offs in cost, comfort, or sustainability.

Current wearable sensors, however, exhibit multiple limitations. They often fail to conform to skin curvature and movement, reducing wear comfort; they depend on chemical adhesives or tapes that may irritate skin or trigger allergies; their adhesion diminishes in humid or sweaty conditions, limiting long-term use; many require external power or auxiliary equipment, constraining portability and practicality; and complex fabrication and limited durability hinder large-scale deployment [11].

Biomimetics provides a promising pathway to overcome these constraints by learning from nature’s evolutionarily optimized designs [12]. The goal is not mere imitation of form, but understanding how organisms solve problems and translating those mechanisms into engineering. For example, lotus leaves exhibit self-cleaning through superhydrophobicity, dragonfly wings rupture bacteria via nanoscale pillars, and bird feathers change color with humidity. Cephalopods adjust skin pigmentation for rapid, reversible color modulation, while pangolins combine rigid scales with flexible joints for protection and mobility [13,14,15,16,17,18]. These cases show that biological systems routinely achieve multifunctionality—adhesion, sensing, antifouling, and mechanical robustness—using minimal energy. Comparative analyses further reveal recurring design rules such as hierarchical structuring, graded stiffness, and stimuli-responsive materials that can inform next-generation sensor design.

Nature’s strategies are informative at multiple levels—form (structure), process (manufacture), and ecosystem (circularity). Form-level strategies can be directly transposed into functional structures; process-level strategies inspire low-energy, low-pollution fabrication; and ecosystem-level strategies guide the integration of products into sustainable, circular systems. Whereas conventional engineering often assumes high energy and material input, biological strategies deliver multifunctionality with minimal resources and reduced environmental burden.

Wearable sensors are a particularly apt domain to showcase biomimetic potential. These devices must adhere comfortably to skin for extended periods, operate with little to no external power, resist fouling and microbial contamination over long-term use, and endure continuous bending and motion—all requirements that are difficult to satisfy simultaneously with conventional approaches but are commonplace in nature.

Accordingly, this study does not present experimental data. Instead, it classifies and analyzes biological mechanisms by function and proposes a conceptual framework for applying them to wearable environmental sensor design. Focusing on four core functions—adhesion, color change, antifouling, and flexible protection—we organize representative cases and integrate them into a multi-layer sensor architecture. The framework advances a platform capable of simultaneously achieving skin-friendly wearability, power-free visual sensing, long-term antifouling performance, and durability under large curvature and repeated deformation, while supporting implementation of the GBF and NbS. The framework connected biological mechanisms across scales, from microstructural interfaces to macroscopic architectures, and translates them into design logics for multilayer wearable sensors. It thus provided a coherent reasoning path linking functional principles, material selection, and system integration for future bioinspired sensor design.

## 2. Functional Classification of Biological Mechanisms and Biomimetic Application

### 2.1. Adhesion Mechanisms: Nature’s Strategies Tailored to Skin and Environment

Skin-mounted wearables must adhere reliably without causing discomfort, while maintaining performance in the presence of sweat and humidity [19]. Conventional reliance on chemical adhesives or tapes risks irritation or allergy and often fails under wet conditions. Nature offers several instructive strategies [20,21]. As summarized in Table 1, the studies collectively illustrated how biological adhesion mechanisms from insect tarsi to snail mucus informed the engineering of skin-compatible dry and wet adhesives based on hierarchical and viscoelastic design principles.

Beetles develop tarsal structures bearing hundreds of thousands of fine tenent setae. Each seta terminates in a broadened spatulate tip that maximizes contact area, enhancing van der Waals interactions and enabling robust adhesion. These structures also help displace interfacial moisture, supporting stable attachment under humid conditions [26]. Many insects further secrete hydrophobic, viscous fluids in micron-scale droplets that augment adhesion and evaporate slowly, sustaining long-term contact [27,28,29,30,31,32]. Snail mucus is a gel composed of polysaccharides and glycoproteins. Its viscoelasticity changes markedly with water content and ionic strength, enabling it to partially harden when dry for temporary fixation, while remaining mobile and strongly adhesive on rough surfaces when wet. Importantly, the mucus can be removed without damage, allowing repeated attachment and detachment [24,33,34,35].

Recent microfabrication studies demonstrate that hierarchical pillar arrays patterned on elastomeric substrates can replicate the combined capillary and van der Waals forces observed in insect tarsi, providing quantitative evidence that biological adhesion principles can be scaled to human skin [19,36]. Integrating humidity-responsive polymers within such structures may further enable “smart” adhesion that strengthens in moist conditions and weakens under dry conditions, offering on-demand detachment without chemical solvents [25]. Collectively, the strategies outlined design rules for skin-friendly and durable adhesion combining insect-inspired microstructured interface with snail-inspired viscoelastic gel [37]. Building upon these biological insights, recent advances now show that skin-adhesive patches are maturing toward scalable manufacturing—combining printable dry/wet adhesives and hydrogel interfaces that maintain adhesion under perspiration and motion—thus enabling long-wear medical and sensing patches at pilot-line scale [38]. Beetle-inspired adhesive systems illustrate how hierarchical micro-setae achieve reversible, residue-free attachment through controlled contact mechanics, offering stronger yet gentler adhesion than conventional pressure-sensitive adhesives [39,40].

### 2.2. Color Change Mechanisms: Harnessing Light and Environment for Passive Sensing

When environmental changes can be communicated via color, sensors can convey information intuitively without external power. Nature has evolved multiple mechanisms that express humidity, temperature, and illumination through color change (Table 2) [41,42,43,44]. The studies demonstrated that humidity and temperature-dependent color shifts in beetles, feathers, and cephalopods were experimentally translated into photonic-hydrogel films and polymeric composites for passive, power-free optical sensing.

Pinecone scales exhibit hygromorphic behavior driven by layer-dependent cellulose microfibril orientations and lignin content. Differential moisture uptake causes anisotropic expansion: outer layers contract upon drying to open the scales, while inner layers swell when humid to close them. This dead-tissue mechanism provides a passive, maintenance-free humidity response [45,46,52]. In some scarab beetles, porous exoskeletal photonic structures form three-dimensional crystals. Under dry conditions, the refractive-index contrast between air and chitin yields bright structural colors; when humidity rises, water fills the pores, reducing index contrast and diminishing reflected color—an archetypal humidity-dependent structural coloration [47]. Feathers such as those of bronzed swallows contain periodically arranged melanosomes in a keratin matrix; hydration alters layer thickness and interference conditions, shifting reflected wavelength and inducing visible color changes [53]. Cephalopods combine pigmentary chromatophores with tunable Bragg-reflector iridophores built from reflectin protein assemblies and broadband leucophores, enabling rapid, reversible, neurally modulated color change that can be harnessed as a dynamic indicator [51,54,55]. Beyond visual appeal, these colorimetric mechanisms offer quantitative sensing potential. For example, humidity-induced shifts in photonic bandgaps of beetle exoskeletons can be correlated with absolute relative humidity, allowing direct optical calibration [56,57]. Similarly, temperature-dependent reflectin phase transitions in cephalopods can yield reversible, wavelength-specific responses within physiologically relevant ranges, suggesting routes to self-powered thermal indicators [47]. Incorporating these biological architectures into multilayer polymer films or hydrogel composites may produce wearable patches that visually report microclimate variations without electronic circuitry [7]. Recent photonic-hydrogel devices translate humidity and analyte-induced bandgap shifts into smartphone-readable color, validating low-power optical patches as manufacturable sensing modules [42,58]. In engineering translation, photonic hydrogel systems highlight how structural coloration was tuned by environmental stimuli without external power [59]. The reversible and rapid optical response made them particularly effective for visual feedback in wearable sensors, where interpretability and low energy demand were more critical than absolute color intensity or response speed [60,61].

### 2.3. Antifouling and Antibacterial Mechanisms: Anti-Contamination and Microbes

Wearables are continuously exposed to contaminants, oils, and microbes that degrade performance. Biological surfaces address these risks through specialized structures [62,63]. Lotus- and Nepenthes-inspired superhydrophobic and lubricant-infused surfaces, together with nanostructured insect wings, provided complementary antifouling and bactericidal performance validated on polymer substrates for wearable use (Table 3). For clarity, antifouling refers to the prevention of nonspecific adhesion of contaminants, proteins, or biofilms, whereas antibacterial denotes the active inhibition or elimination of microbial growth. Although the mechanisms were often coupled in biological surfaces, they are treated here as distinct but complementary functions. The epidermis of lotus leaf features micropapillae overlain by nanoscale wax crystals, a hierarchical texture that minimizes solid–liquid contact area; the resulting superhydrophobicity yields high contact angles and low hysteresis, causing rolling droplets to remove dust and debris (self-cleaning) [64,65]. The inner surface of Nepenthes comprises a porous cellular scaffold infused with lubricating liquid held by capillarity; the persistent thin film produces slippery, low-adhesion interfaces across diverse liquids, inspiring slippery liquid-infused porous surfaces (SLIPS) with anti-biofouling performance [66,67,68]. Arrays of nanoscale pillars on insect wings can inactivate bacteria by mechanically stressing cell envelopes; recent work shows additional roles for penetration and oxidative stress pathways in their antibacterial activity, providing antibiotic-free strategies that mitigate resistance concerns [69,70,71]. For wearable sensors, these strategies can be combined to deliver multi-modal protection. Superhydrophobic coatings minimize sweat accumulation and particulate adhesion, SLIPS-inspired layers prevent sebum fouling, and mechano-bactericidal nanostructures provide passive antimicrobial defense [72]. Recent in vivo tests on polymer-based SLIPS films indicate stable performance over several weeks of continuous skin contact, highlighting their translational potential for long-term health monitoring devices. For optically read wearables, transparent liquid-infused (SLIPS/LIS) coatings preserve clarity while resisting fouling and microbial attachment, supporting stable colorimetric readouts under sweat and sebum. In addition, mechano-bactericidal and self-cleaning textures compatible with skin devices can be replicated over large areas via nanoimprint lithography, which continues to scale toward industrial roll-to-roll throughput. On flexible, skin-mounted substrates, liquid-infused antifouling coatings can maintain interfacial stability—and, when paired with optically clear elastic supports, preserve transparency—under repeated bending and perspiration exposure; practically, durability hinges on lubricant retention and compliant underlayers rather than specific endurance metrics [73].

### 2.4. Flexible Protective Architectures: Natural Armor That Balances Stiffness and Compliance

Skin-mounted devices experience continuous bending, pressure, and repetitive motion (Table 4) [77]. They must withstand external impacts while deforming naturally with the skin—an apparent trade-off between stiffness and flexibility [78,79,80]. Recent investigations of pangolin and armadillo scale architectures confirm how graded stiffness and segmented geometry can be reproduced in soft-matrix composites to achieve flexible yet impact-resistant protection.

Overlapping keratinous scales in Pangolin scales layer to distribute impact forces while flexible skin between scales preserves mobility, enabling animals to curl and perform varied movements—simultaneously achieving protection and compliance [81,85]. Banded bony plates of Armadillo are connected via cartilaginous joints, maintaining a rigid exterior while permitting rolling mobility. The architecture balances plate stiffness with joint compliance to resist external pressure and repetitive deformation. These exoskeletal designs inspire protective layers for wearables that maintain device integrity without sacrificing conformability on highly curved, mobile skin regions [86]. Analogous design has been demonstrated in segmented elastomer–ceramic composites, where overlapping tiles embedded in a stretchable matrix yield high puncture resistance without restricting tensile strain. Finite-element modeling further shows that graded joint thickness and interplate curvature can be tuned to distribute stress uniformly, reducing the risk of delamination under repeated bending. Such insights can guide the fabrication of outer layers that protect sensing elements during daily activity or accidental impacts. Scale-inspired segmented shells now demonstrate programmable stiffness and shape on soft substrates, pointing to protective overlays that absorb impact without sacrificing conformability in wearable form factors. Collectively, these perspectives extend the biological cases into functional design concepts, clarifying how natural mechanisms can inform material selection, structural organization, and performance stability in wearable sensor engineering.

## 3. Proposed Integrated Sensor Architecture

Although adhesion, color change, antifouling, and flexible protection appear as separate functions, practical wearable environmental sensors required their simultaneous integration within a coherent system [87]. To complement the qualitative discussion, Table 5 summarized the representative biological mechanisms, their engineering interpretations, and relative performance benchmarks. It also schematically illustrated how these functions are hierarchically organized within the multilayer sensor architecture, thereby reinforcing the engineering relevance of the framework. At the system level, printed/transfer-assembled body-conformable electronics and near-field wireless links provide a practical route to multilayer stacks that co-integrate adhesion, passive optical transduction, antifouling skins, and protective shells for continuous on-skin operation [88,89]. Such devices must adhere comfortably, operate visually with minimal power, resist environmental contaminants, and tolerate daily bending and impact [90]. The proposed multilayer sensor architecture integrated four biological functions—adhesion, color change, antifouling, and flexible protection—within a unified framework. Stable integration required material compatibility to prevent layer delamination and maintain flexibility under repeated use [91]. Optical transparency was preserved through optimized protective coatings that balance transmittance and durability [92]. Moreover, an amphiphilic buffer layer harmonized strong skin adhesion with antifouling performance [93]. These considerations outlined a feasible route for combining the four biomimetic functions into a single, nature-integrated sensor system. These requirements are best addressed through a layered architecture extending outward from the skin interface. Adhesive interface is low-irritation, reversible adhesion inspired by insect microsetae and snail-mucus gels [94]. Sensing and display layer is power-free colorimetric response inspired by pinecone hygromorphs, humidity-tunable beetle photonics and cephalopod-like dynamic color modulation [95,96]. Antifouling and antimicrobial layer is self-cleaning, bactericidal surface based on lotus-leaf microtextures, liquid-infused porosity, and dragonfly-wing nanostructures. The protective outer layer consists of segmented, flexible shells resembling pangolin and armadillo scales to ensure impact resistance without sacrificing flexibility.

Collectively, the tables serve to comparatively visualize how the four biological functions interlock within a multilayered design. The integrative framework not only summarizes cross-scale relationships but also acts as a conceptual map linking natural strategies to engineering translation. These layers are not merely stacked; they are co-designed to be mutually supportive. The adhesive layer is optimized in thickness and refractive properties so as not to interfere with optical and colorimetric readouts. The antifouling layer preserves color fidelity and long-term stability by minimizing surface contamination and biofilm formation. The protective layer shields sensing elements and interconnects from cyclic bending and incidental impacts, extending service life. This biologically grounded, layer-by-layer integration offers a practical design starting point for harmonizing skin compatibility, power-free responsiveness, antifouling stability, and durability under high curvature [105,106,107]. To transition from conceptual design to manufacturable prototypes, additional parameters—such as interlayer diffusion of moisture, compatibility of fabrication temperatures, and recyclability of composite materials—must be systematically evaluated. Advanced additive manufacturing, including multi-material 3D printing and soft lithography, offers promising routes to integrate these biologically inspired features within a single production workflow. Pilot studies combining photonic hydrogels with SLIPS coatings demonstrated simultaneous humidity sensing and antifouling capability, offering proof-of-concept evidence that multi-layer biomimetic integration is technically feasible. The co-design perspective highlights synergistic relationships among layers, with adhesion enhancing optical stability, antifouling maintaining signal fidelity, and flexible protection preserving mechanical integrity, illustrating how functional reciprocity defines the holistic performance of biomimetic wearable systems.

## 4. Conclusions

This study reinterprets functional and structural mechanisms observed in biological surfaces within a function-oriented framework and integrates them into layer-specific design strategies for skin-interfaced environmental sensors. By systematically categorizing natural mechanisms into four functional domains—adhesion, color change, antifouling, and flexible protection—it demonstrates how biodiversity-informed principles can be translated into a coherent engineering framework. This approach bridges biological understanding and materials science, establishing a conceptual foundation for developing sensor platforms that are both sustainable and high-performing.

From an application perspective, recent advances in bioinspired adhesives, photonic hydrogels, and antifouling coatings highlight the growing feasibility of practical implementation. Several studies indicate that biomimetic materials can be fabricated through scalable and adaptable processes, signaling a gradual transition from conceptual design toward real-world deployment. This convergence between biological principles and engineering design underscores the evolution of biomimetics from descriptive inspiration to functional, nature-integrated technology.

The novelty of this study lies in its explicit synthesis across multiple levels—functional classification, hierarchical sensor architecture, and sustainability alignment. By translating biodiversity-informed strategies into an integrated, multilayer design paradigm, this framework moves beyond conventional biomimetic reviews and provides a tangible pathway toward nature-integrated engineering consistent with the objectives of the Global Biodiversity Framework and Nature-based Solutions.

Future research directions included quantitative validation and process optimization. Key directions include evaluating the balance between adhesion strength and skin compatibility under dynamic, sweat-rich conditions; establishing calibration standards for humidity- and temperature-responsive colorimetric systems; ensuring the long-term stability and recyclability of antifouling coatings; and analyzing mechanical durability and moisture diffusion across integrated layers. These investigations will not only determine technical feasibility but also clarify environmental implications throughout the product life cycle, ensuring that biomimetic wearable sensors progress toward scalable, sustainable, and socially beneficial technologies.

## Figures and Tables

**Table 1:** Biological adhesion mechanisms and engineering implications.

Organism	Structure/Material	Functional Characteristic	Implication for Sensor Design	Ref.
Beetle	Microsetae + secreted fluid	Stable adhesion in humid conditions; repeatable attachment	Conformal, low-irritation adhesion on curved skin	[22,23]
Snail	Glycoprotein–polysaccharide mucus	Tunable viscoelasticity; reversible detachment	Long-term wear with minimal skin damage	[24,25]

**Table 2:** Color change mechanisms and sensor applications.

Organism	Mechanism	Key Feature	Sensor Application	Ref.
Pinecone	Hygromorphic expansion/contraction	Passive humidity response	Humidity-responsive indicator	[45,46]
Beetle	Porous photonic nanostructure	Humidity-dependent structural color shift	Power-free optical humidity sensor	[47,48]
Swallow feather	Periodic melanosome interference	Hydration-dependent color change	Environmental colorimetric indicator	[49,50]
Squid	Chromatophores + reflectin-based iridophores	Rapid, reversible color modulation	Intuitive, power-free display	[7,51]

**Table 3:** Distinct mechanisms and applications of antifouling and antibacterial surfaces.

Organism	Structure	Function	Implication for Sensors	Ref.
Lotus leaf	Micro/nano hierarchical roughness	Self-cleaning superhydrophobicity	Reduced contamination; long-term stability	[64,74]
Nepenthes	Liquid-infused porous surface	Omniphobic, low adhesion	Resistance to diverse liquid fouling	[68,75]
Dragonfly wing	Nanoscale pillar arrays	Physical bactericidal action	Hygiene without antibiotics	[71,76]

**Table 4:** Flexible protective mechanisms and applications.

Organism	Structure	Feature	Sensor Application	Ref.
Pangolin	Overlapping keratin scales	Impact absorption with flexibility	Shock-resistant, conformal device layer	[81,82]
Armadillo	Banded osteoderms + cartilaginous joints	Rigidity and compliance	Durable, skin-adaptive protection	[83,84]

**Table 5:** Layered integration of biological functions for wearable sensor design.

Function	Representative Organisms	Core Feature	Role in Sensor Architecture	Ref.
Adhesion	Beetle; Snail	Stable, repeatable attachment via microsetae and viscoelastic gel	Low-irritation, reversible adhesion maintained in humid conditions	[97,98]
Color change	Pinecone; Beetle; Swallow feather; Squid	Humidity/temperature/neural control of color	Power-free visual sensing and display	[99,100]
Antifouling	Lotus; Nepenthes; Dragonfly	Self-cleaning, lubricated omniphobicity, bactericidal nano-topography	Protection from contamination/microbes; stabilized signals	[101,102]
Flexible protection	Pangolin; Armadillo	Coexistence of rigid plates and compliant joints	Conformal durability; impact resistance	[103,104]

## Data Availability

No new data were created or analyzed in this study. Data sharing is not applicable to this article.

## References

[1] IPCC (2023). Synthesis Report of the IPCC Sixth Assessment Report (AR6).

[2] IPBES (2019). Global Assessment Report on Biodiversity and Ecosystem Services. Bonn: Intergovernmental Science-Policy Platform on Biodiversity and Ecosystem Services.

[3] CBD (2022). Kunming–Montreal Global Biodiversity Framework.

[4] Heikenfeld J., Jajack A., Rogers J., Gutruf P., Tian L., Pan T., Li R., Khine M., Kim J., Wang J. (2018). Wearable sensors: Modalities, challenges, and prospects. Lab Chip.

[5] Gao W., Emaminejad S., Nyein H.Y.Y., Challa S., Chen K., Peck A., Fahad H.M., Ota H., Shiraki H., Kiriya D. (2016). Fully integrated wearable sensor arrays for multiplexed in situ perspiration analysis. Nature.

[6] Someya T., Bao Z., Malliaras G.G. (2016). The rise of plastic bioelectronics. Nature.

[7] Lin Y.-C., Yang C., Tochikura S., Uzarski J.R., Morse D.E., Sepunaru L., Gordon M.J. (2025). Electrochemically driven optical dynamics of reflectin protein films. Adv. Mater..

[8] Mishra T., Wang M., Metwally A.A., Bogu G.K., Brooks A.W., Bahmani A., Alavi A., Celli A., Higgs E., Dagan-Rosenfeld O. (2020). Pre-symptomatic detection of COVID-19 from smartwatch data. Nat. Biomed. Eng..

[9] Quer G., Radin J.M., Gadaleta M., Baca-Motes K., Ariniello L., Ramos E., Kheterpal V., Topol E.J., Steinhubl S.R. (2021). Wearable sensor data and self-reported symptoms for COVID-19 detection. Nat. Med..

[10] Natarajan A., Su H.-W., Heneghan C. (2020). Assessment of physiological signs associated with COVID-19 measured using wearable devices. NPJ Digit. Med..

[11] Kim J., Campbell A.S., de Ávila B.E.-F., Wang J. (2019). Wearable biosensors for healthcare monitoring. Nat. Biotechnol..

[12] Bhushan B. (2009). Biomimetics: Lessons from nature—An overview. Philos. Trans. R. Soc. A..

[13] Barthlott W., Neinhuis C. (1997). Purity of the sacred lotus, or escape from contamination in biological surfaces. Planta.

[14] Ivanova E.P., Hasan J., Webb H.K., Gervinskas G., Juodkazis S., Truong V.K., Wu A.H.F., Lamb R.N., Baulin V.A., Watson G.S. (2013). Bactericidal activity of black silicon. Nat. Commun..

[15] Shawkey M.D., Morehouse N.I., Vukusic P. (2009). A protean palette: Colour materials and mixing in birds and butterflies. J. R. Soc. Interface.

[16] Mäthger L.M., Denton E.J., Marshall N.J., Hanlon R.T. (2009). Mechanisms and behavioural functions of structural coloration in cephalopods. J. R. Soc. Interface.

[17] Wang B., Yang W., Sherman V.R., Meyers M.A. (2016). Pangolin armor: Overlapping, structure, and mechanical properties of the keratinous scales. Acta Biomater..

[18] Chen I.H., Kiang J.H., Correa V., Lopez M.I., Chen P.-Y., McKittrick J., Meyers M.A. (2011). Armadillo armor: Mechanical testing and micro-structural evaluation. J. Mech. Behav. Biomed. Mater..

[19] Guo Y., Wang X., Zhang L., Zhou X., Wang S., Jiang L., Chen H. (2025). From dry to wet, the nature inspired strong attachment surfaces and their medical applications. ACS Nano.

[20] Al-Amri A.M. (2025). Printed sensors for environmental monitoring: Advancements, challenges, and future directions. Chemosensors.

[21] Albu C., Chira A., Radu G.-L., Eremia S.A.V. (2025). Advances in cost-effective chemosensors for sustainable monitoring in food safety and processing. Chemosensors.

[22] Li M., Mao A., Guan Q., Saiz E. (2024). Nature-inspired adhesive systems. Chem. Soc. Rev..

[23] Song J.H., Baik S., Kim D.W., Yang T.-H., Pang C. (2021). Wet soft bio-adhesion of insect-inspired polymeric oil-loadable perforated microcylinders. Chem. Eng. J..

[24] Lai J.H., del Alamo J.C., Rodríguez-Rodríguez J., Lasheras J.C. (2010). The mechanics of the adhesive locomotion of terrestrial gastropods. J. Exp. Biol..

[25] Kang M., Sun K., Seong M., Hwang I., Jang H., Park S., Choi G., Lee S.-H., Kim J., Jeong H.E. (2021). Applications of bioinspired reversible dry and wet adhesives: A review. Front. Mech. Eng..

[26] Baik S., Lee J., Jeon E.J., Park B.-y., Kim D.W., Song J.H., Lee H.J., Han S.Y., Cho S.-W., Pang C. (2021). Diving beetle–like miniaturized plungers with reversible, rapid biofluid capturing for machine learning–based care of skin disease. Sci. Adv..

[27] Federle W. (2006). Why are so many adhesive pads hairy?. J. Exp. Biol.

[28] Dirks J.-H., Federle W. (2011). Fluid-based adhesion in insects—Principles and challenges. Soft Matter.

[29] Arzt E., Gorb S., Spolenak R. (2003). From micro to nano contacts in biological attachment devices. Proc. Natl. Acad. Sci. USA.

[30] Peisker H., Michels J., Gorb S.N. (2013). Evidence for a material gradient in the adhesive tarsal setae of the ladybird beetle *Coccinella septempunctata*. Nat. Commun..

[31] Kovalev A.E., Filippov A.E., Gorb S.N. (2013). Insect wet steps: Loss of fluid from insect feet adhering to a substrate. J. R. Soc. Interface.

[32] Chen Y., Zhu Z., Steinhart M., Gorb S.N. (2022). Bio-inspired adhesion control with liquids. iScience.

[33] Denny M. (1980). The role of gastropod pedal mucus in locomotion. Nature.

[34] Smith A.M. (2002). The structure and function of adhesive gels from invertebrates. Integr. Comp. Biol.

[35] Deng T., Gao D., Song X., Zhou Z., Zhou L., Tao M., Jiang Z., Yang L., Luo L., Zhou A. (2023). A natural biological adhesive from snail mucus for wound repair. Nat. Commun..

[36] Shi Z., Tan D., Liu Q., Meng F., Zhu B., Xue L. (2021). Tree frog-inspired nanopillar arrays for enhancement of adhesion and friction. Biointerphases.

[37] Hou C., He W., Yao X. (2025). Mucus-inspired supramolecular adhesives: Exploring the synergy between dynamic networks and functional liquids. ACS Nano.

[38] Wong S.H.D., Deen G.R., Bates J.S., Maiti C., Lam C.Y.K., Pachauri A., AlAnsari R., Bělský P., Yoon J., Dodda J.M. (2023). Smart skin-adhesive patches: From design to biomedical applications. Adv. Funct. Mater..

[39] Wan X., Wang Z., Liu M., Zhang F., Wang S. (2024). Bioinspired structural adhesives: A decades-old science but emerging materials. Matter.

[40] Tripathi D., Ray P., Singh A.V., Kishore V., Singh S.L. (2023). Durability of slippery liquid-infused surfaces: Challenges and advances. Coatings.

[41] Butt M.A., Piramidowicz R. (2024). Integrated photonic sensors for the detection of toxic gasses—A review. Chemosensors.

[42] Bravo-Yagüe J.C., Paniagua-González G., Garcinuño R.M., García-Mayor A., Fernández-Hernando P. (2025). Colorimetric molecularly imprinted polymer-based sensors for rapid detection of organic compounds: A review. Chemosensors.

[43] Basso C.R., Filho M.V.B., Gavioli V.D., Parra J.P.R.L.L., Castro G.R., Pedrosa V.A. (2025). Recent advances in nanomaterials for enhanced colorimetric detection of viruses and bacteria. Chemosensors.

[44] Meng X., Zhang R., Dong X., Wang Z., Yu L. (2025). A pH-responsive liquid crystal-based sensing platform for the detection of biothiols. Chemosensors.

[45] Cheng T., Tahouni Y., Sahin E.S., Ulrich K., Lajewski S., Bonten C., Wood D., Rühe J., Speck T., Menges A. (2024). Weather-responsive adaptive shading through biobased and bioinspired hygromorphic 4D-printing. Nat. Commun..

[46] Ulrich K., Mylo M.D., Masselter T., Scheckenbach F., Fischerbauer S., Nopens M., Flenner S., Greving I., Hesse L., Speck T. (2025). Beyond the bilayer: Multilayered hygroscopic actuation in pine cone scales. Beilstein J. Nanotechnol..

[47] Sun J., Wu W., Tian L., Li W., Zhang F., Wang Y. (2021). Investigation of the selective color-changing mechanism of *Dynastes tityus beetle* (Coleoptera: Scarabaeidae). Sci. Rep..

[48] Wei H., Chen C., Yang D. (2024). Applications of inverse opal photonic crystal hydrogels in the preparation of acid-base color-changing materials. RSC Adv..

[49] Rashid I., Hassan M.U., Nazim M., Elsherif M., Dou Q., Hu D., Kamran M., Dai Q., Butt H. (2020). Structural colouration in the *Himalayan monal*, hydrophobicity and refractive index modulated sensing. Nanoscale.

[50] Freyer P., Wilts B.D., Stavenga D.G. (2021). Cortex thickness is key for the colors of iridescent starling feather barbules with a single, organized melanosome layer. Front. Ecol. Evol..

[51] Wolde-Michael E., Roberts A.D., Heyes D.J., Dumanli A.G., Blaker J.J., Takano E., Scrutton N.S. (2021). Design and fabrication of recombinant reflectin-based multilayer reflectors: Bio-design engineering and photoisomerism induced wavelength modulation. Sci. Rep..

[52] Reyssat E., Mahadevan L. (2009). Hygromorphs: From pine cones to biomimetic bilayers. J. R. Soc. Interface.

[53] Noh H., Liew S.F., Saranathan V., Mochrie S.G.J., Prum R.O., Dufresne E.R., Cao H. (2010). How noniridescent colors are generated by quasi-ordered structures of bird feathers. Adv. Mater..

[54] DeMartini D.G., Krogstad D.V., Morse D.E. (2013). Membrane invaginations facilitate reversible water flux driving tunable iridescence in a dynamic biophotonic system. Proc. Natl. Acad. Sci. USA.

[55] Ghoshal A., Demartini D.G., Eck E., Morse D.E. (2013). Optical parameters of the tunable Bragg reflectors in squid. J. R. Soc. Interface.

[56] Jung S.-H., Lee H.T., Park M.J., Lim B., Park B.C., Jung Y.J., Kong H., Hwang D.-H., Lee H.-i., Park J.M. (2021). Precisely tunable humidity color indicator based on photonic polymer films. Macromolecules.

[57] Kim J.H., Moon J.H., Lee S.-Y., Park J. (2010). Biologically inspired humidity sensor based on three-dimensional photonic crystals. Appl. Phys. Lett..

[58] Qin J., Wang W., Cao L. (2023). Photonic hydrogel sensing system for wearable and noninvasive cortisol monitoring. ACS Appl. Polym. Mater..

[59] Xu W., Ma Z., Tian Q., Chen Y., Jiang Q., Fan L. (2025). A review of fluorescent pH probes: Ratiometric strategies, extreme pH sensing, and multifunctional utility. Chemosensors.

[60] Ko B., Jeon N., Kim J., Kang H., Seong J., Yun S., Badloe T., Rho J. (2024). Hydrogels for active photonics. Microsyst. Nanoeng..

[61] Choe A., Yeom J., Shanker R., Kim M.P., Kang S., Ko H. (2018). Stretchable and wearable colorimetric patches based on thermoresponsive plasmonic microgels embedded in a hydrogel film. NPG Asia Mater..

[62] Vasilescu A., Gáspár S., Gheorghiu M., Polonschii C., Banciu R.M., David S., Gheorghiu E., Marty J.-L. (2025). Promising solutions to address the non-specific adsorption in biosensors based on coupled electrochemical-surface plasmon resonance detection. Chemosensors.

[63] Xiao G., Li J., Zhao B., Yue Z. (2025). Photoelectrochemical aptasensors for biosensing: A review. Chemosensors.

[64] Tu W., Luo Y., Shen J., Ran X., Yu Z., Wang C., Cai C., Bi H. (2025). Lotus leaf-inspired corrosion-resistant and robust superhydrophobic coating for oil-water separation. Biomimetics.

[65] Wang Y., Li J., Song H., Wang F., Su X., Zhang D., Xu J. (2025). Biomimetic superhydrophobic surfaces: From nature to application. Materials.

[66] Wong T.-S., Kang S.H., Tang S.K.Y., Smythe E.J., Hatton B.D., Grinthal A., Aizenberg J. (2011). Bioinspired self-repairing slippery surfaces with pressure-stable omniphobicity. Nature.

[67] Yang Y., Zhu Q., Xu L.P., Zhang X. (2022). Bioinspired liquid-infused surface for biomedical and biosensing applications. Front. Bioeng. Biotechnol..

[68] Jia Y., Yang Y., Cai X., Zhang H. (2024). Recent developments in slippery liquid-infused porous surface coatings for biomedical applications. ACS Biomater. Sci. Eng..

[69] Pogodin S., Hasan J., Baulin V., Webb H., Truong V.K., Nguyen H.P., Boshkovikj V., Fluke C., Watson G., Watson J. (2013). Biophysical model of bacterial cell interactions with nanopatterned cicada wing surfaces. Biophys. J..

[70] Jenkins J., Mantell J., Neal C., Gholinia A., Verkade P., Nobbs A.H., Su B. (2020). Antibacterial effects of nanopillar surfaces are mediated by cell impedance, penetration and induction of oxidative stress. Nat. Commun..

[71] Jin E., Lv Z., Zhu Y., Zhang H., Li H. (2024). Nature-inspired micro/nano-structured antibacterial surfaces. Molecules.

[72] Eichler-Volf A., Xue L., Kovalev A., Gorb E.V., Gorb S.N., Steinhart M. (2016). Nanoporous monolithic microsphere arrays have anti-adhesive properties independent of humidity. Molecules.

[73] Shen J., Ou J., Lei S., Hu Y., Wang F., Fang X., Li C., Li W., Amirfazli A. (2023). Innovative solid slippery coating: Uniting mechanical durability, optical transparency, anti-Icing, and anti-graffiti traits. Polymers.

[74] Chen Y., Jiao Z., Jiao S., Quan Z., Zhang C., Li B., Niu S., Han Z., Ren L. (2025). Bio-inspired anti-glare and self-cleaning surface for optical/tissue residue pollution treatment of medical device surface. Chem. Eng. J..

[75] Etim I.-I.N., Zhang R., Wang C., Khan S., Mathivanan K., Duan J. (2025). New insights on slippery lubricant-infused porous surfaces technique in mitigating microbial corrosion. NPJ Mater. Degrad..

[76] Tan N., Im J., Neate N., Leong C.-O., Wildman R.D., Marsh G.E., Yee M.S.-L. (2024). Mechano-bactericidal activity of two-photon polymerized micro-and nanoscale topographies against Pseudomonas aeruginosa: Surface interactions and antibacterial efficacy. Mater. Today Commun..

[77] Machín A., Márquez F. (2025). Next-generation chemical sensors: The convergence of nanomaterials, advanced characterization, and real-world applications. Chemosensors.

[78] Pantano A., Baiamonte V. (2025). Mechanics of bio-inspired protective scales. Biomimetics.

[79] Ghods S., Murcia S., Ossa E.A., Arola D. (2019). Designed for resistance to puncture: The dynamic response of fish scales. J. Mech. Behav. Biomed. Mater..

[80] Chen T., Yang X., Zhang B., Li J., Pan J., Wang Y. (2024). Scale-inspired programmable robotic structures with concurrent shape morphing and stiffness variation. Sci. Robot..

[81] Lazarus B.S., Chadha C., Velasco-Hogan A., Barbosa J.D.V., Jasiuk I., Meyers M.A. (2021). Engineering with keratin: A functional material and a source of bioinspiration. iScience.

[82] Choi J., Han S., Baliwag M., Kim B.H., Jang H., Kim J.-T., Hong I., Kim T., Kang S.M., Lee K.-T. (2022). Artificial stretchable armor for skin-interfaced wearable devices and soft robotics. Extrem. Mech. Lett..

[83] Teeple J.B., Cohen K.E., Stankowich T., Paig-Tran E.W.M., Donatelli C.M. (2025). Tapered tiles modulate flexibility in segmented armadillo-inspired armor. Integr. Comp. Biol..

[84] Lu Y., Zhao D., He J., Zou L. (2023). Armadillo-inspired ultra-sensitive flexible sensor for wearable electronics. Chem. Eng. J..

[85] Liu Q., Mao H., Niu L., Chen F., Ma P. (2022). Excellent flexibility and stab-resistance on pangolin-inspired scale-like structure composite for versatile protection. Compos. Commun..

[86] Connors M., Yang T., Hosny A., Deng Z., Yazdandoost F., Massaadi H., Eernisse D., Mirzaeifar R., Dean M.N., Weaver J.C. (2019). Bioinspired design of flexible armor based on chiton scales. Nat. Commun..

[87] Ates H.C., Nguyen P.Q., Gonzalez-Macia L., Morales-Narváez E., Güder F., Collins J.J., Dincer C. (2022). End-to-end design of wearable sensors. Nat. Rev. Mater..

[88] Wei R., Li H., Chen Z., Hua Q., Shen G., Jiang K. (2024). Revolutionizing wearable technology: Advanced fabrication techniques for body-conformable electronics. NPJ Flex. Electron..

[89] Tang J., Li R., Mahmood S., Li J., Yao S. (2025). Carbon nanotube-based chemical sensors: Sensing mechanism, functionalization and applications. Chemosensors.

[90] Wang P., Xu S., Shi X., Zhu J., Xiong H., Wen H. (2025). Recent advances in resistive gas sensors: Fundamentals, material and device design, and intelligent applications. Chemosensors.

[91] Bauer M., Duerkop A., Baeumner A.J. (2023). Critical review of polymer and hydrogel deposition methods for optical and electrochemical bioanalytical sensors correlated to the sensor’s applicability in real samples. Anal. Bioanal. Chem..

[92] Won D., Bang J., Choi S.H., Pyun K.R., Jeong S., Lee Y., Ko S.H. (2023). Transparent electronics for wearable electronics application. Chem. Rev..

[93] Maan A.M.C., Hofman A.H., de Vos W.M., Kamperman M. (2020). Recent developments and practical feasibility of polymer-based antifouling coatings. Adv. Funct. Mater..

[94] Ben Halima H., Lakard B., Jaffrezic-Renault N. (2025). Microneedle-based sensors for wearable diagnostics. Chemosensors.

[95] Kou D., Zhang S., Ma W. (2024). Recent advances in 1D photonic crystals: Diverse morphologies and distinctive structural colors for multifaceted applications. Adv. Opt. Mater..

[96] Bogdanov G., Strzelecka A.A., Kaimal N., Senft S.L., Lee S., Hanlon R.T., Gorodetsky A.A. (2025). Gradient refractive indices enable squid structural color and inspire multispectral materials. Science.

[97] Zhou Z., Deng T., Tao M., Lin L., Sun L., Song X., Gao D., Li J., Wang Z., Wang X. (2023). Snail-inspired AFG/GelMA hydrogel accelerates diabetic wound healing via inflammatory cytokines suppression and macrophage polarization. Biomaterials.

[98] Tian H., Wang D., Zhang Y., Jiang Y., Liu T., Li X., Wang C., Chen X., Shao J. (2022). Core–shell dry adhesives for rough surfaces via electrically responsive self-growing strategy. Nat. Commun..

[99] Bandodkar A.J., Lee S.P., Huang I., Li W., Wang S., Su C.J., Jeang W.J., Hang T., Mehta S., Nyberg N. (2020). Sweat-activated biocompatible batteries for epidermal electronic and microfluidic systems. Nat. Electron..

[100] Song J.W., Ryu H., Bai W., Xie Z., Vázquez-Guardado A., Nandoliya K., Avila R., Lee G., Song Z., Kim J. (2023). Bioresorbable, wireless, and battery-free system for electrotherapy and impedance sensing at wound sites. Sci. Adv..

[101] Li J., Zhou Z., Jiao X., Guo Z., Fu F. (2024). Bioinspired lubricant-infused porous surfaces: A review on principle, fabrication, and applications. Surf. Interfaces.

[102] Muneer M., Kalappurackal H.P., Balachandran A., Lone S. (2024). Biological design and inspiration of bactericidal hierarchical interfaces. RSC Appl. Interfaces.

[103] Zhang F., Zhu P., Lu P., Qian K., Liu S., Wang L. (2024). Biomimetic design and impact simulation of Al_2_O_3_/Al composite armor based on armadillo shell. Sci. Rep..

[104] Karami Fath I., Niknejad A., Zare-Zardini H. (2025). Design optimization of pangolin inspired composites for enhanced energy absorption. Sci. Rep..

[105] IUCN (2020). Global Standard for Nature-Based Solutions: A User-Friendly Framework for the Verification, Design and Scaling up of NbS.

[106] Koh A., Kang D., Xue Y., Lee S., Pielak R.M., Kim J., Hwang T., Min S., Banks A., Bastien P. (2016). A soft, wearable microfluidic device for the capture, storage, and colorimetric sensing of sweat. Sci. Transl. Med..

[107] Kim D.-H., Lu N., Ma R., Kim Y.-S., Kim R.-H., Wang S., Wu J., Won S.M., Tao H., Islam A. (2011). Epidermal electronics. Science.

